# Antibiotic Resistance Patterns of *Pseudomonas* spp. Isolated from the River Danube

**DOI:** 10.3389/fmicb.2016.00586

**Published:** 2016-05-03

**Authors:** Clemens Kittinger, Michaela Lipp, Rita Baumert, Bettina Folli, Günther Koraimann, Daniela Toplitsch, Astrid Liebmann, Andrea J. Grisold, Andreas H. Farnleitner, Alexander Kirschner, Gernot Zarfel

**Affiliations:** ^1^Institute of Hygiene, Microbiology and Environmental Medicine, Medical University GrazGraz, Austria; ^2^Institute of Molecular Biosciences, University of GrazGraz, Austria; ^3^Interuniversity Cooperation Centre for Water and HealthVienna, Austria; ^4^Research Group Environmental Microbiology and Molecular Ecology, Institute of Chemical Engineering, Vienna University of TechnologyVienna, Austria; ^5^Institute for Hygiene and Applied Immunology, Medical University of ViennaVienna, Austria

**Keywords:** JDS3, *Pseudomonas*, antibiotic resistance, water, Danube

## Abstract

Spread and persistence of antibiotic resistance pose a severe threat to human health, yet there is still lack of knowledge about reservoirs of antibiotic resistant bacteria in the environment. We took the opportunity of the Joint Danube Survey 3 (JDS3), the world's biggest river research expedition of its kind in 2013, to analyse samples originating from different sampling points along the whole length of the river. Due to its high clinical relevance, we concentrated on the characterization of *Pseudomonas* spp. and evaluated the resistance profiles of *Pseudomonas* spp. which were isolated from eight sampling points. In total, 520 *Pseudomonas* isolates were found, 344 (66.0%) isolates were identified as *Pseudomonas putida*, and 141 (27.1%) as *Pseudomonas fluorescens*, all other *Pseudomonas* species were represented by less than five isolates, among those two *P. aeruginosa* isolates. Thirty seven percent (37%) of all isolated *Pseudomonas* species showed resistance to at least one out of 10 tested antibiotics. The most common resistance was against meropenem (30.4%/158 isolates) piperacillin/tazobactam (10.6%/55 isolates) and ceftazidime (4.2%/22 isolates). 16 isolates (3.1%/16 isolates) were multi-resistant. For each tested antibiotic at least one resistant isolate could be detected. Sampling points from the upper stretch of the River Danube showed more resistant isolates than downriver. Our results suggest that antibiotic resistance can be acquired by and persists even in *Pseudomonas* species that are normally not in direct contact with humans. A possible scenario is that these bacteria provide a reservoir of antibiotic resistance genes that can spread to related human pathogens by horizontal gene transfer.

## Introduction

Multiresistant bacteria are present in many surface waters (Girlich et al., 2011; Czekalski et al., 2012; Tissera and Lee, 2013; Blaak et al., 2015; Maravic et al., 2015). Typically, evidence is provided through fecal indicators, and mostly relates to short river sections or sampling at individual points. Thus, investigations of whole water systems are rare, especially if the river passes through 10 riparian countries, like the River Danube. Water samples from the third Joint Danube Survey (JDS3), the world's biggest river research expedition of its kind, offered a chance for evaluating resistance of bacteria over a whole river system. Based on these samples, a resistance profile of *Pseudomonas* spp. over the course of the multinational River Danube was created in our study.

*Pseudomonas* species can be naturally found in all surface waters, lakes and rivers, but they are rarely found in drinking water. *Pseudomonas* spp. can survive in both low and high nutrition environments (Mena and Gerba, 2009) or even in double distilled water and, in addition, can help Salmonellae survive in this environment (Warburton et al., 1994). The whole group of non-fermenting Gram negative bacilli is suspected of establishing the basis for multiresistance in Gram negative bacteria, as the members of this group carry multiple intrinsic resistances and have the ability to acquire and evolve additional resistances (Farinas and Martinez-Martinez, 2013). *Pseudomonas* species are known to harbor multiple intrinsic and acquired resistance genes, host several mobile genetic elements, and also exchange them with other families of Gram negative bacilli like *Enterobacteriaceae* (Juan Nicolau and Oliver, 2010; Pfeifer et al., 2010). Hence *Pseudomonas* are known starting points of several important carbapenemases families (Pfeifer et al., 2010). The occurrence and spread of carbapenemases have become a substantial global health problem, as they inactivate a substantial antibiotic class.

The most common pathogen in this genus is *Pseudomonas aeruginosa*. It causes a variety of different infections, from easy-to-cure ear infections, serious infections of burn patients, to severe lung infections which lead to major complications in cystic fibrosis patients (Barbier and Wolff, 2010; Azzopardi et al., 2014). Besides *Pseudomonas aeruginosa*, other species e.g., *Pseudomonas putida* or *Pseudomonas fluorescens* are also a cause for infections in clinical settings (Gilarranz et al., 2013; Erol et al., 2014; Bhattacharya et al., 2015; Mazurier et al., 2015).

The aim of the study was to evaluate the resistance profiles of *Pseudomonas* spp. isolated at selected sites along the whole course of the River Danube. *Pseudomonas* spp. were chosen for various reasons: they belong to the native bacterial community in surface waters, they are clinically relevant, and changes in their natural resistance profiles indicate anthropogenic influence. This study, therefore, aims at monitoring the presence of resistances of *Pseudomonas* spp. to clinically important antibiotics along the river course. Doing so, changes in the resistance profiles were to be detected, if possible.

## Materials and methods

### Sample collection

Samples were collected during the JDS 3, which was organized by the International Commission for the Protection of the Danube River (ICPDR). The ICPDR is a transnational body, which has been established to implement the Danube River Protection Convention. All Danube countries are member states of the ICPDR on the base of the “Convention on Cooperation for the Protection and Sustainable use of the Danube River” (Danube River Protection Convention).

Between Aug. 12 and Sep. 26, 2013, surface water samples for microbiological investigations were collected from 68 sampling sites along the river Danube (JDS 3, 2015).

**Figure 1:**
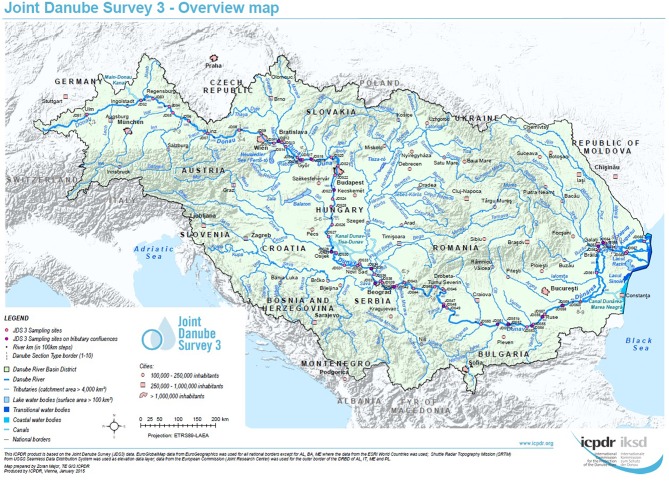
**Overview of the JDS3 sampling points along the river Danube**. The map was taken with kind permission of the ICPDR. (http://www.danubesurvey.org/results).

For each sampling site, water samples were taken at three sampling points, on the left, in the middle and on the right side of the River Danube. Samples were collected in sterile 1-L glass flasks, from 30 cm below the river surface (Figure 1, pink and violet dots, high resolution map is added as Supplementary Figure S1). Duplicate volumes of the samples (45 ml) were filled into sterile non-toxic 50 ml plastic vials (Techno Plastic Products AG, TPP, Switzerland), containing 5 ml glycerol (final conc. 10% v/v). The vials were shaken and turned around to homogenize glycerol and water and after that immediately stored at −20°C on board of the JDS3 research ship. After transfer to the home laboratory (beginning of October 2013) the samples were stored at −80°C. Out of the 68 sampling sites the four sites directly downstream from the cities Vienna, Budapest, Novi Sad, and Bucharest were chosen for investigation. In addition four non-city related sampling sites were chosen (including the delta and near the starting point of the JDS3; Table 1).

**Table 1:** **Investigated sampling sites names and numbers according to JDS3, country and detailed location (ds = downstream, us = upstream)**.

					***E. coli* [MPN/100ml]**		
**Site**	**Name**	**Country**	**Location**	**rkm**	**Left**	**Middle**	**Right**
JDS03	Geisling	Germany	ds Regensburg	2354	1728	1739	1304
JDS10	Wildungsmauer	Austria	ds Vienna	1895	1044	917	1739
JDS22	DS Budapest	Hungary	ds Budapest	1632	4320	6792	6310
JDS28	US Drava	Croatia/Serbia	us tributary Drava	1384	2445	1998	2880
JDS36	DS Tisa, Serbia	Serbia	ds Novi Sad	1200	11060	9900	4960
JDS59	DS Arges	Romania/Bulgary	ds tributary Arges^*^	429	288000	720	1983
JDS63	Siret	Romania	Galati	154	7565	11851	7120
JDS68	St. Gheorge arm	Romania	river delta	104	4880	2424	2431

*Basic microbiological parameters. MPN, most probable numbers; rkm, river kilometer. E. coli concentrations were determined according to ISO 9308-2 (JDS 3, 2015)*.

* *Arges is the river flowing through Bucharest*.

### Isolation of bacteria

The frozen samples were thawed and 15 ml (left, middle, right 5 ml each) were plated in 0.5 ml portions on different selective agars: Endo Agar, Xylose Lysine Deoxycholat Agar (XLD agar), and Chromocult Coliform Agar (CCA), (all Merck, Austria). Growth conditions were 37 ± 1°C for 18–24 h. Identification of Pseudomonas by MALDI-TOF-MS (matrix-assisted laser desorption ionization time-of-flight mass spectrometry) was per-formed as described previously (Jamal et al., 2014). A single bacterial colony was deposited on the target slide, followed by the addition of the matrix (VITEK MS-CHCA) and air drying. Samples were processed in the MALDI-TOF-MS spectrometer VITEK® MS (Biomerieux, Austria). Microbial identification was achieved by obtaining the spectra using MALDI-TOF technology and analyzing the spectra with the VITEK MS database. The peaks from these spectra were compared with the characteristic pattern for the species, genus or family of the microorganism, leading to identification of the organism.

### Antibiotic susceptibility testing

For all identified *Pseudomonas* spp., antibiotic susceptibility testing was performed as recommended by the European Committee on Antimicrobial Susceptibility Testing (EUCAST) including recommended controls. Inhibition zone diameters were interpreted according to EUCAST guidelines (http://www.eucast.org/clinical_breakpoints/) (EUCAST, 2013; Matuschek et al., 2014). Classification of multiresistance of *Pseudomonas* spp. was evaluated according to the Robert Koch Institut (RKI, Germany, http://www.rki.de/EN/Home/homepage_node.html). The suspension for inoculation was prepared from an over-night pure culture on a blood agar (non-selective medium). Colonies were picked with a sterile loop and suspended in sterile saline (0.85% NaCl w/v in water) to the density of a McFarland 0.5 standard (DensiCheck, Biomerieux, Austria). The suspension was then plated on Mueller-Hinton Agar by using an automatic plate rotator (Retro C80, Biomerieux, Austria). Antibiotic test disks were applied firmly on the agar surface within 15 min of inoculation of the plates. Plates were incubated at 36°C for 16–20 h. After incubation, inhibition zones were measured.

The following antibiotics were tested:

Piperacilin/tazobactam (TZP), ceftazidime (CAZ), cefepime (FEP), meropenem (MEM), imipenem (IPM), amikacin (AN), gentamicin (GM), tobramycin (NN), ciprofloxacin (CIP), levofloxacin (LEV), and sulfamethoxazole/trimethoprim (SXT) (all Becton Dickinson, Schwechat, Austria; Table 2).

**Table 2:** **EUCAST Clinical Breakpoints for ***Pseudomonas*** spp**.

**Antibiotic**	**Antibiotic centration on test disk (μg)**	**Susceptibly inhibition zone (mm)**	**Resistant (including intermediate) inhibition zone (mm)**
Piperacilin/tazobactam	30/6	≥18	<18
Ceftazidime	10	≥16	<16
Cefepime	30	≥19	<19
Meropenem	10	≥18	<18
Imipenem	10	≥20	<20
Amikacin	30	≥24	<24
Gentamicin	10	≥15	<15
Tobramycin	10	≥16	<16
Ciprofloxacin	5	≥25	<25
Levofloxacin	5	≥20	<20

SXT was evaluated because sulfamethoxazole was part of the chemical analysis of the River Danube water. There are no sulfamethoxazole/trimethoprim breakpoints according to EUCAST for *Pseudomonas* spp. To include the sensitivity of *Pseudomonas* spp. to sulfamethoxazole/trimethoprim, diameters of inhibition areas were evaluated and compared (SXT test discs specification: sulfamethoxazole/trimethoprim: 1.25/23.75 μg).

### Modified hodge test

To estimate the presence of carbapenemases, a modified Hodge test was performed with all isolates resistant to at least one carbapenem. In brief: after plating of a carbapenem sensitive *Klebsiella pneuomoniae* (ATCC 700603) a 10 μg imipenem or meropenem disc was placed in the center, and each test isolate was streaked from the disk to the edge of the plate. After incubation (37 ± 1°C for 18–24 h) the plates were checked for showing a “cloverleaf shaped” inhibition zone. Isolates that produced carbapenemases enabled growth of the sensitive Klebsiella closer to the antibiotic disk (Bennett et al., 2009).

### Data analyses

Statistical analyses were calculated with GraphPadPrism^TM^ 5.01 for Windows, GraphPad Software, San Diego California USA, www.graphpad.com.

## Results

### Species composition of isolates

In total, 520 *Pseudomonas* spp. were isolated, the fewest isolates were obtained from JDS68 (32 isolates), and the highest number could be isolated from JDS28 sample with 117 isolates (Table 3). The most abundant *Pseudomonas* species were *Pseudomonas putida* (66.0%/344 isolates) and *Pseudomonas fluorescens* (27.1%/141 isolates). Each of the other detected species represented less than 1% of all isolates (five or fewer isolates). Only two *Pseudomonas aeruginosa* were isolated, both from JDS28.

**Table 3:** **Number of isolated ***Pseudomonas*** spp. at the investigated sampling points (SP) and the number of resistant isolates to the testes antibioitic**.

**SP**	**TZP**	**CAZ**	**FEP**	**MEM**	**IPM**	**AN**	**GM**	**NN**	**CIP**	**LEV**	**No. Isolates**
JDS03	3	10	1	15	0	0	1	0	9	1	35
JDS10	2	2	0	16	2	0	0	0	1	0	33
JDS22	15	5	0	32	6	1	0	0	2	0	46
JDS28	19	4	2	35	3	0	0	0	2	3	117
JDS36	6	0	0	10	0	1	1	1	1	1	109
JDS59	6	1	1	21	0	0	0	0	0	0	46
JDS63	3	0	0	21	0	0	0	0	2	1	102
JDS68	1	0	0	8	0	0	0	0	1	0	32
Sum	55	22	4	158	11	2	2	1	18	6	520

*Tested antibiotics and their abbreviation: (TZP), piperacilin/tazobactam; (CAZ), ceftazidime; (FEP), cefepime; (MEM), meropenem; (IPM), imipenem; (AN), amikacin; (GM), gentamicin; (NN), tobramycin; (CIP), ciprofloxacin; (LEV), levofloxacin*.

### Antibiotic resistances

Wild type *Pseuodomas* species are susceptible to all tested antibiotics except SXT (EUCAST, 2013). The highest number of resistances was the one against meropenem with 158 resistant isolates (30.4%), 55 isolates (10.6%) were resistant to piperacilin/tazobactam and 22 isolates (4.2%) resistant to ceftazidime, 11 (2.1%) to imipenem and four (0.8%) to cefepime (Table 3). In the fluoroquinolone group only six (1.2%) of the isolates showed levofloxacin resistance, whereas 18 (3.4%) isolates were resistant against ciprofloxacin. Resistance to ciprofloxacin turned out to be the most frequent resistance of all tested non beta-lactam antibiotics in this study. One isolate which was resistant to levofloxacin was still sensitive to ciprofloxacin. Resistance to aminoglycosides was very rare, two isolates were resistant to amikacin and gentamicin respectively and only one isolate showed no susceptibility to tobramycin (Table 3). The 11 isolates resistant to imipenem were also resistant to meropenem and were positive in the modified Hodge test, indicating carbapenemase activity. 327 (62.9%) isolates were susceptible to all tested antibiotics with EUCAST breakpoints (clinical resistance wild type), 128 (24.6%) isolates showed resistance to one, and 49 (9.4%) isolates were resistant to two tested antibiotics. The most common combination of resistances was to meropenem and piperacillin/tazobactam. Sixteen isolates revealed resistance to 3 or more antibiotics, including 12 (2.3%) isolates with three resistances, three (0.6%) isolates with four and one (0.2%) with five. Eight of them were classified as multi-resistant as they were resistant to three or four different antibiotic classes (Table 4).

**Table 4:** **Resistance pattern of isolates showing resistances to three or more of the tested antibiotics**.

**Isolate**	**Species**	**Resistance Pattern**	**Multidrug Resistance (MDR)**
JDS03PS007	*Pseudomonas fluorescens*	CAZ, MEM, CIP	MDR 3
JDS03PS016	*Pseudomonas fluorescens*	TZP, CAZ, GM, MEM, CIP	MDR 4
JDS03PS019	*Pseudomonas putida*	TZP, MEM, CIP	MDR 3
JDS03PS020	*Pseudomonas fluorescens*	CAZ, MEM, CIP	MDR 3
JDS03PS032	*Pseudomonas fluorescens*	CAZ, CIP, LEV	
JDS22PS016	*Pseudomonas putida*	TZP, CAZ, MEM	MDR 3
JDS22PS018	*Pseudomonas fluorescens*	CAZ, IMP, MEM	
JDS22PS032	*Pseudomonas putida*	TZP, IMP, MEM	
JDS22PS035	*Pseudomonas putida*	TZP, IMP, MEM	
JDS22PS043	*Pseudomonas putida*	TZP, MEM, CIP	MDR 3
JDS28PS083	*Pseudomonas fluorescens*	CAZ, FEP, IMP, MEM	
JDS28PS113	*Pseudomonas fluorescens*	CAZ, IMP, MEM	
JDS28PS115	*Pseudomonas putida*	TZP, MEM, CIP, LEV	MDR 3
JDS28PS117	*Pseudomonas putida*	TZP, MEM, LEV	MDR 3
JDS36PS036	*Pseudomonas putida*	TZP, AN, GM, NN	
JDS59PS020	*Pseudomonas fluorescens*	TZP, CAZ, FEP	

*Multidrug resistance was assigned to an isolate if it revealed a resistance to three (MDR 3) or four (MDR 4) antibiotic classes. Antibiotic classes: acylureidopenicillins: (TZP) piperacilin/tazobactam; cephalosporins: (CAZ) ceftazidime, (FEP) cefepime; carbapenems: (MEM) meropenem, (IPM) imipenem; aminoglycosides: (AN) amikacin, (GM) gentamicin, (NN) tobramycin; fluoroquinolones: (CIP) ciprofloxacin, (LEV) levofloxacin*.

The 12 isolates with three antibiotic resistances split in eight different resistance patterns, the isolates with four resistances all displayed the same pattern. The two *Pseudomonas aeruginosa* were susceptible to all tested antibiotics. Out of all other isolated species in this study at least one showed the EUCAST defined antibiotic susceptibility wild type, susceptible to all tested antibiotics (except SXT; Supplementary Table S1).

Four sampling points had less than 50% isolates with clinical resistance wild type, JDS03, JDS10, JDS22, and JDS59. Three sampling points revealed no isolate that was non-susceptible to three or more tested antibiotics, JDS10, JDS63 and JDS68 (Table 4).

**Figure 2:**
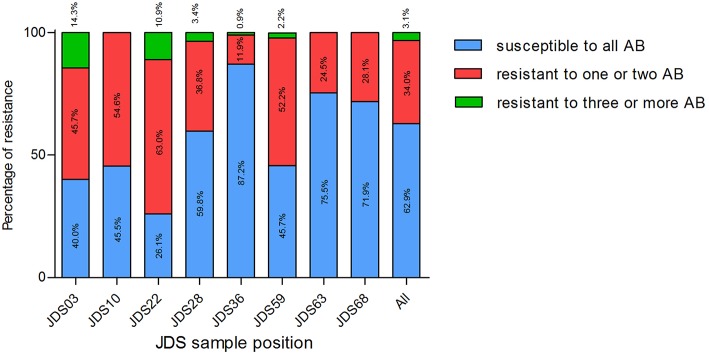
**Proportion of isolates susceptible to all tested antibiotics (blue bars), resistant to one or two tested antibiotics (red bars) and resistant to three or more tested antibiotics (green bars), at different sampling points**. JDS03, JDS28, JDS63, and JDS68 are non-urban sampling sites, whereas JDS10 is ds Vienna, JDS22 ds Budapest, JDS36 ds Novi Sad and JDS59 is ds Bucharest. Kruskal-Wallis test revealed a non-Gaussian distribution with a *p* < 0.0001.

Surprisingly, the upstream sampling points (JDS03, JDS10, JDS22, and JDS28) revealed higher proportions of resistant bacteria than the downstream ones (Figure 2). Sampling point JDS03 revealed the highest proportions of resistance to ceftazidime (28.6% of JDS03 isolates) and ciprofloxacin (25.7% of JDS03 isolates). Sampling point JDS22 showed high rates for piperacillin/tazobactam (32.6% of the JDS22 isolates), meropenem (69.6% of the JDS22 isolates), and imipenem (13.0% of the JDS22 isolates).

**Figure 3:**
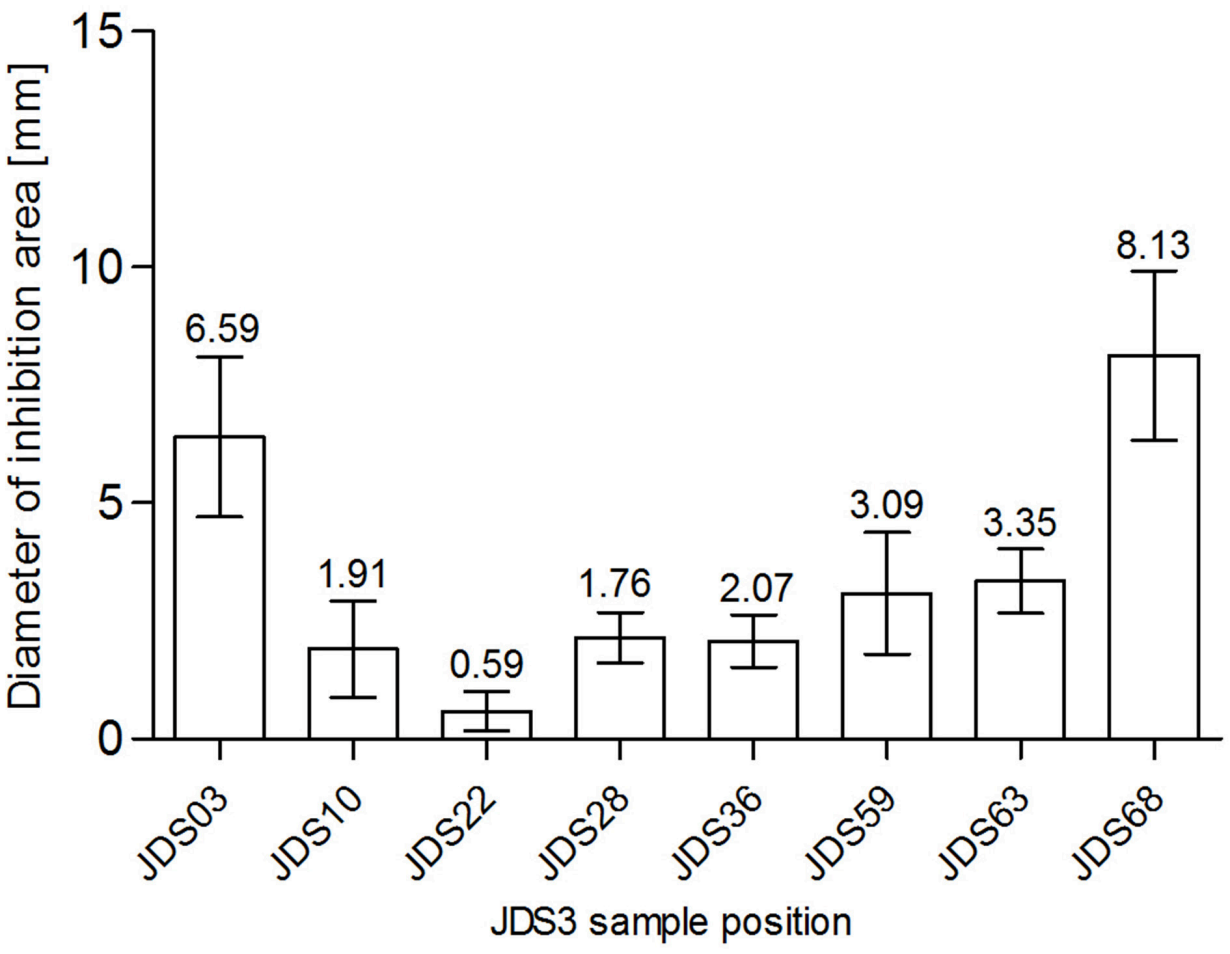
**Zone of inhibition diameters (mm) for SXT at the investigated sampling sites**. Bars show mean values (values given on top of the bars, mm) and standard deviation errors bars (SD error bars) for each sampling site.

SXT was chosen for testing as sulfamethoxazole was measured directly during JDS3. Chemical analysis revealed a sampling site (JDS58) with elevated levels for sulfamethoxazole (Arges, tributary) (JDS 3, 2015). The subsequent River Danube sampling site JDS59 did not show significantly reduced diameters (*p* = 0.68) (Figure 3). JDS03 and JDS68 showed both elevated diameters (6.59 mm JDS03, 8.13 mm JDS68) but only JDS68 differed significantly from the other sampling points (*p* < 0.05).

## Discussion

The spread of antibiotic resistant bacteria, their distribution, and their reservoirs in the environment are important issues. Within the last years many different possible sources have been intensively investigated to shed light on the spread of antibiotic resistant bacteria. Farming and the spread of liquid manure are known to contribute to the spread of multiresistant bacteria (Sengupta et al., 2011; Friese et al., 2013). Recently, the focus has been put on waste water, as bacteria of all kind and with all possible genetic features are mixed up there. And a very critical feature in waste water is the possibility that bacteria harboring resistance exchange their resistance determinants with other bacteria (Korzeniewska and Harnisz, 2013; Reinthaler et al., 2013; Amador et al., 2015). Microorganisms from these and other sources can be relatively easily flushed into surface waters (Czekalski et al., 2012; Zurfluh et al., 2013; Hess and Gallert, 2014), but except for a few studies on relatively small rivers that deal with this topic, the fate of deposited bacteria is quite unclear.

The distribution of susceptible and resistant *Pseudomonas* spp. at the investigated JDS3 sampling points showed site-specific differences. At the upstream sampling points, there was a trend to more resistant bacteria (JDS03, JDS10, JDS22, JDS28), and multiresistant *Pseudomonas* spp. could only be detected in this part of the river. One reason for this finding could be the lower river water volume in the upstream parts of the River Danube, which might result in less dilution of the resistant microorganisms. Downstream from the cities of Vienna, Budapest, and Bucharest (JDS10, JDS22, and JDS59 via Argeş; Figure 3), the occurrence of resistant bacterial isolates was also elevated, with anthropogenic influence very likely being the reason for that.

Under similar non-selective isolation conditions Suzuki et al. found no resistance to meropenem, gentamicin, amikacin, or ciprofloxacin (Suzuki et al., 2013). A presence of only 8 (1.5%) multiresistant *Pseudomonas* spp. present in three of the eight sampling points seems to be a low number. But still, if we extrapolate the number of 8 multiresistant *Pseudomonaceae* in a collected volume of 75 ml at the 5 sampling points (5 × 15 ml) to 1 liter, we might estimate over 100 multiresistant bacteria of the *Pseudomonas* group in one liter of Danube water.

The isolates all over the course of the river Danube showed high resistance rates against meropenem (9.2–69.6%). The resistance against carbapenems in *Pseudomonas* spp. is mostly mediated via efflux pumps (intrinsic resistance), especially in water environment (Tacao et al., 2015). However, carbapenem resistance poses a challenge for therapy, regardless of the underlying mechanism. For example, *Pseudomonas putida* (66% of all isolates in this study) is increasingly involved in hospital infections (Kim et al., 2012; Molina et al., 2014). These infections come up with severe complications and high mortality rates (up to 40%). In most of these cases multiresistant *Pseudomonas putida* was the reason for the infection or the nosocomial outbreak (Kim et al., 2012).

This study is the first study investigating bacterial resistance in a transnational river survey (2500 rkm). Although it was limited by a small sample volume and a fixed time course, the results of this study substantiate the occurrence of waterborne *Pseudomonas* spp. with non-wild type resistance pattern in the whole River Danube. Their presence and their distribution suggest human influence.

## Author contributions

CK study design, laboratory work, manuscript preparation, data analysis, ML laboratory work, data conservation. RB laboratory work. BF laboratory work. GK data analysis, manuscript preparartion. DT laboratory work, data analysis. AL laboratory work. AG manuscript preparation. AF manuscript preparation. AK sample collection, manuscript preparation. GZ study design, laboratory work, data analysis, manuscript preparation.

## Funding

The Joint Danube Survey was organized by the International Commission for the Protection of the Danube River (ICPDR). The study was supported by the Austrian Science Fund (FWF), project nr P25817-B22.

### Conflict of interest statement

The authors declare that the research was conducted in the absence of any commercial or financial relationships that could be construed as a potential conflict of interest.

## Supplementary material

Supplementary Figure S1 **Figure of the JDS3 River Danube Survey (high resolution.pdf)**.

Supplementary Table S1 **Table of all *Pseudomonas* spp. isolates with resistance pattern**. (TZP), Piperacilin/tazobactam; (CAZ), ceftazidime; (FEP), cefepime; (MEM), meropenem; (IPM), imipenem; (AN), amikacin; (GM), gentamicin; (NN), tobramycin; (CIP), ciprofloxacin; (LEV), levofloxacin; and (SXT), sulfamethoxazole/trimethoprim.

## References

[B1] Amador P. P. Fernandes R. M. Prudencio M. C. Barreto M. P. Duarte I. M. (2015). Antibiotic resistance in wastewater: occurrence and fate of Enterobacteriaceae producers of class A and class C beta-lactamases. J. Environ. Sci. Health. A. Tox. Hazard. Subst. Environ. Eng. 50, 26–39. 10.1080/10934529.2015.964602 25438129

[B2] Azzopardi E. A. Azzopardi E. Camilleri L. Villapalos J. Boyce D. E. Dziewulski P.. (2014). Gram negative wound infection in hospitalised adult burn patients–systematic review and metanalysis-. PLoS ONE 9:e95042. 10.1371/journal.pone.0095042 24751699 PMC3994014

[B3] Barbier F. Wolff M. (2010). Multi-drug resistant *Pseudomonas aeruginosa*: towards a therapeutic dead end? Med. Sci. (Paris) 26, 960–968. 10.1051/medsci/20102611960 21106178

[B4] Bennett J. W. Herrera M. L. Lewis J. S. II. Wickes B. W. Jorgensen J. H. (2009). KPC-2-producing Enterobacter cloacae and pseudomonas putida coinfection in a liver transplant recipient. Antimicrob. Agents Chemother. 53, 292–294. 10.1128/AAC.00931-08 18852270 PMC2612145

[B5] Bhattacharya D. Dey S. Kadam S. Kalal S. Jali S. Koley H.. (2015). Isolation of NDM-1-producing multidrug-resistant Pseudomonas putida from a paediatric case of acute gastroenteritis, India. New Microbes New Infect. 5, 5–9. 10.1016/j.nmni.2015.02.002 25893095 PMC4398820

[B6] Blaak H. Lynch G. Italiaander R. Hamidjaja R. A. Schets F. M. de Roda Husman A. M. (2015). Multidrug-resistant and extended spectrum beta-lactamase-producing *Escherichia coli* in Dutch Surface Water and Wastewater. PLoS ONE 10:e0127752. 10.1371/journal.pone.0127752 26030904 PMC4452230

[B7] Czekalski N. Berthold T. Caucci S. Egli A. Burgmann H. (2012). Increased levels of multiresistant bacteria and resistance genes after wastewater treatment and their dissemination into lake geneva, Switzerland. Front. Microbiol. 3:106. 10.3389/fmicb.2012.00106 22461783 PMC3310248

[B8] Erol S. Zenciroglu A. Dilli D. Okumus N. Aydin M. Gol N.. (2014). Evaluation of nosocomial blood stream infections caused by Pseudomonas species in newborns. Clin. Lab. 60, 615–620. 10.7754/Clin.Lab.2013.130325 24779295

[B9] EUCAST (2013). European Committee on Antimicrobial Susceptibility Testing (EUCAST). Växjö.

[B10] Farinas M. C. Martinez-Martinez L. (2013). Multiresistant Gram-negative bacterial infections: Enterobacteria, *Pseudomonas aeruginosa, Acinetobacter baumannii* and other non-fermenting Gram-negative bacilli. Enferm. Infecc. Microbiol. Clin. 31, 402–409. 10.1016/j.eimc.2013.03.016 23684390

[B11] Friese A. Schulz J. Laube H. von Salviati C. Hartung J. Roesler U. (2013). Faecal occurrence and emissions of livestock-associated methicillin-resistant Staphylococcus aureus (laMRSA) and ESbl/AmpC-producing E. coli from animal farms in Germany. Berl. Munch. Tierarztl. Wochenschr. 126, 175–180. 10.2376/0005-9366-126-175 23540202

[B12] Gilarranz R. Juan C. Castillo-Vera J. Chamizo F. J. Artiles F. Alamo I.. (2013). First detection in Europe of the metallo-beta-lactamase IMP-15 in clinical strains of *Pseudomonas putida* and *Pseudomonas aeruginosa*. Clin. Microbiol. Infect. 19, E424–E427. 10.1111/1469-0691.12248 23656535

[B13] Girlich D. Poirel L. Nordmann P. (2011). Diversity of clavulanic acid-inhibited extended-spectrum beta-lactamases in Aeromonas spp. from the Seine River, Paris, France. Antimicrob. Agents Chemother. 55, 1256–1261. 10.1128/AAC.00921-10 21149627 PMC3067085

[B14] Hess S. Gallert C. (2014). Demonstration of staphylococci with inducible macrolide-lincosamide-streptogramin B (MLSB) resistance in sewage and river water and of the capacity of anhydroerythromycin to induce MLSB. FEMS Microbiol. Ecol. 88, 48–59. 10.1111/1574-6941.12268 24308503

[B15] Jamal W. Albert M. J. Rotimi V. O. (2014). Real-time comparative evaluation of bioMerieux VITEK MS versus Bruker Microflex MS, two matrix-assisted laser desorption-ionization time-of-flight mass spectrometry systems, for identification of clinically significant bacteria. BMC Microbiol. 14:289-014-0289-0. 10.1186/s12866-014-0289-0 25433488 PMC4290442

[B16] JDS 3 I. (2015). Joint Danube Survey 3. A Comprehensive Analysis of Danube Water Quality. Vienna: ICPDR – International Commission for the Protection of the Danube River.

[B17] Juan Nicolau C. Oliver A. (2010). Carbapenemases in Pseudomonas spp. Enferm. Infecc. Microbiol. Clin. 28 (Suppl. 1), 19–28. 10.1016/S0213-005X(10)70004-5 20172419

[B18] Kim S. E. Park S. H. Park H. B. Park K. H. Kim S. H. Jung S. I.. (2012). Nosocomial Pseudomonas putida Bacteremia: high rates of carbapenem resistance and mortality. Chonnam Med. J. 48, 91–95. 10.4068/cmj.2012.48.2.91 22977749 PMC3434797

[B19] Korzeniewska E. Harnisz M. (2013). Extended-spectrum beta-lactamase (ESBL)-positive Enterobacteriaceae in municipal sewage and their emission to the environment. J. Environ. Manage. 128, 904–911. 10.1016/j.jenvman.2013.06.051 23886578

[B20] Maravic A. Skocibusic M. Cvjetan S. Samanic I. Fredotovic Z. Puizina J. (2015). Prevalence and diversity of extended-spectrum-beta-lactamase-producing Enterobacteriaceae from marine beach waters. Mar. Pollut. Bull. 90, 60–67. 10.1016/j.marpolbul.2014.11.021 25480155

[B21] Matuschek E. Brown D. F. Kahlmeter G. (2014). Development of the EUCAST disk diffusion antimicrobial susceptibility testing method and its implementation in routine microbiology laboratories. Clin. Microbiol. Infect. 20, O255–O266. 10.1111/1469-0691.12373 24131428

[B22] Mazurier S. Merieau A. Bergeau D. Decoin V. Sperandio D. Crepin A. Barbey C.. (2015). Type III secretion system and virulence markers highlight similarities and differences between human- and plant-associated pseudomonads related to *Pseudomonas fluorescens* and *P. putida*. Appl. Environ. Microbiol. 81, 2579–2590. 10.1128/aem.04160-14 25636837 PMC4357948

[B23] Mena K. D. Gerba C. P. (2009). Risk assessment of *Pseudomonas aeruginosa* in water. Rev. Environ. Contam. Toxicol. 201, 71–115. 10.1007/978-1-4419-0032-6_3 19484589

[B24] Molina L. Udaondo Z. Duque E. Fernàndez M. Molina-Santiago C. Roca A. Porcel M.. (2014). Antibiotic resistance determinants in a *Pseudomonas putida* strain isolated from a hospital. PLoS ONE 9:e81604. 10.1371/journal.pone.0081604 24465371 PMC3894933

[B25] Pfeifer Y. Cullik A. Witte W. (2010). Resistance to cephalosporins and carbapenems in Gram-negative bacterial pathogens. Int. J. Med. Microbiol. 300, 371–379. 10.1016/j.ijmm.2010.04.005 20537585

[B26] Reinthaler F. F. Galler H. Feierl G. Haas D. Leitner E. Mascher F.. (2013). Resistance patterns of *Escherichia coli* isolated from sewage sludge in comparison with those isolated from human patients in 2000 and 2009. J. Water. Health. 11, 13–20. 10.2166/wh.2012.207 23428545

[B27] Sengupta N. Alam S. I. Kumar R. B. Singh L. (2011). Diversity and antibiotic susceptibility pattern of cultivable anaerobic bacteria from soil and sewage samples of India. Infect. Genet. Evol. 11, 64–77. 10.1016/j.meegid.2010.10.009 20965279

[B28] Suzuki Y. Kajii S. Nishiyama M. Iguchi A. (2013). Susceptibility of *Pseudomonas aeruginosa* isolates collected from river water in Japan to antipseudomonal agents. Sci. Total Environ. 450-451, 148–154. 10.1016/j.scitotenv.2013.02.011 23474260

[B29] Tacao M. Correia A. Henriques I. S. (2015). Low prevalence of carbapenem-resistant Bacteria in River Water: resistance is mostly related to intrinsic mechanisms. Microb. Drug Resist. 21, 497–506. 10.1089/mdr.2015.0072 26430939

[B30] Tissera S. Lee S. M. (2013). Isolation of Extended Spectrum beta-lactamase (ESBL) Producing Bacteria from Urban Surface Waters in Malaysia. Malays. J. Med. Sci. 20, 14–22. 23966820 PMC3743977

[B31] Warburton D. W. Bowen B. Konkle A. (1994). The survival and recovery of *Pseudomonas aeruginosa* and its effect upon salmonellae in water: methodology to test bottled water in Canada. Can. J. Microbiol. 40, 987–992. 10.1139/m94-158 7704834

[B32] Zurfluh K. Hachler H. Nuesch-Inderbinen M. Stephan R. (2013). Characteristics of extended-spectrum beta-lactamase- and carbapenemase-producing Enterobacteriaceae Isolates from rivers and lakes in Switzerland. Appl. Environ. Microbiol. 79, 3021–3026. 10.1128/AEM.00054-13 23455339 PMC3623138

