# Supplementary material for: Antibiotic Resistance Patterns of Pseudomonas spp. Isolated from the River Danube
Source: Front Microbiol. 2016 May 3;7:586. doi: 10.3389/fmicb.2016.00586 (PMC4853796; doi:10.3389/fmicb.2016.00586)
Supplement: Supplementary Figure S1 — Figure of the JDS3 River Danube Survey (high resolution.pdf). [file Image1.PDF]

Joint Danube Survey 3 - Overview map

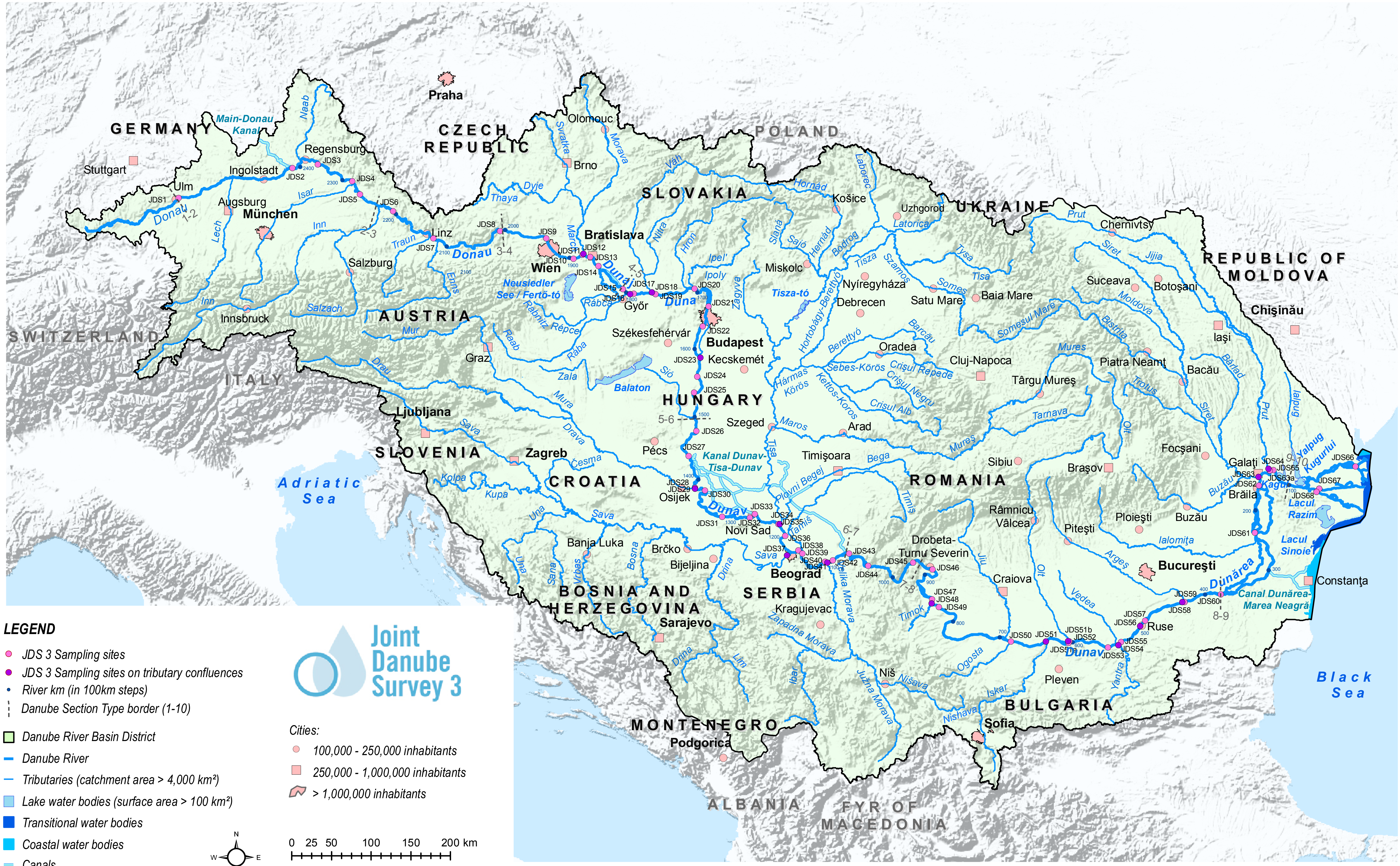

www.icpdr.org

icpdr iksd
International Commission for the Protection of the Danube River
Internationale Kommission zum Schutz der Donau

This ICPDR product is based on the Joint Danube Survey (JDS3) data. EuroGlobalMap data from EuroGeographics was used for all national borders except for AL, BA, ME where the data from the ESRI World Countries was used; Shuttle Radar Topography Mission (SRTM) from USGS Seamless Data Distribution System was used as elevation data layer; data from the European Commission (Joint Research Center) was used for the outer border of the DRBD of AL, IT, ME and PL.

Map prepared by Zoran Major, TE GIS ICPDR
Produced by ICPDR, Vienna, January 2015
